# CasDacGCN: A Dynamic Attention-Calibrated Graph Convolutional Network for Information Popularity Prediction

**DOI:** 10.3390/e27101064

**Published:** 2025-10-14

**Authors:** Bofeng Zhang, Yanlin Zhu, Zhirong Zhang, Kaili Liao, Sen Niu, Bingchun Li, Haiyan Li

**Affiliations:** 1 School of Computer Science and Technology, Kashi University, Kashi 844000, China; bfzhang@sspu.edu.cn (B.Z.);; 2 School of Computer and Information Engineering, Institute for Artificial Intelligence, Shanghai Polytechnic University, Shanghai 201209, Chinaklliao@sspu.edu.cn (K.L.); niusen@sspu.edu.cn (S.N.)

**Keywords:** information popularity prediction, information diffusion, temporal graph, graph convolutional network

## Abstract

Information popularity prediction is a critical problem in social network analysis. With the increasing prevalence of social platforms, accurate prediction of the diffusion process has become increasingly important. Existing methods mainly rely on graph neural networks to model structural relationships, but they are often insufficient in capturing the complex interplay between temporal evolution and local cascade structures, especially in real-world scenarios involving sparse or rapidly changing cascades. To address this issue, we propose the Cascading Dynamic attention-calibrated Graph Convolutional Network, named CasDacGCN. It enhances prediction performance through spatiotemporal feature fusion and adaptive representation learning. The model integrates snapshot-level local encoding, global temporal modeling, cross-attention mechanisms, and a hypernetwork-based sample-wise calibration strategy, enabling flexible modeling of multi-scale diffusion patterns. Results from experiments demonstrate that the proposed model consistently surpasses existing approaches on two real-world datasets, validating its effectiveness in popularity prediction tasks.

## 1. Introduction

In recent years, with the rising impact of social media platforms like Twitter, Weibo, and Facebook, people increasingly use social media to express opinions and share daily experiences. As a result, online public opinion has become a critical factor in social governance and stability. Understanding the diffusion patterns of online content and predicting its future popularity has aroused considerable attention since its wide applications in public management, commercial decisions, and public safety [1,2,3,4,5].

Nevertheless, owing to the public nature of social networks and the massive scale of user interactions, the complex relationships and noisy content present in social media make popularity prediction highly challenging. An information cascade represents the spread of content among users via follow and repost behaviors. A single user’s action may significantly influence the final popularity of a post, making cascade prediction inherently difficult. The goal of cascade popularity prediction is to estimate the future trend derived from observed diffusion history.

One approach involves modeling the dynamic process of information diffusion over networks. If the diffusion pattern can be summarized by a simple model with a few parameters and the underlying network topology is known, the model can be fitted using observed data to predict future trends. However, the complex link structures involved in real-world propagation processes are often beyond the capacity of such simplified models to capture. Generative models, such as Hawkes processes and their variants, have been widely used to model self-exciting diffusion dynamics [6,7,8,9], but they usually assume homogeneous triggering effects and often underperform when cascades exhibit heterogeneous temporal bursts. Another approach adopts traditional machine learning techniques, which predict future trends by feeding selected key features into a predictor [10]. Nevertheless, this method suffers from significant uncertainty and subjectivity in both feature selection and quantification [11].

With the advancement of deep learning, end-to-end models have emerged to bypass manual feature engineering. Many recent works utilize network structures and temporal dynamics as input, as these modalities are platform-independent and generalizable. A growing number of studies combine graph convolutional networks (GCN) for structural encoding [12,13,14,15,16] with RNN for temporal modeling. Despite these advances, most existing studies evaluate on limited observation windows (e.g., 1–3 h on Weibo), rely on fixed GCN aggregation that ignores directional asymmetry in cascades, and seldom address the long-tail phenomenon where the majority of cascades are small and sparse. GCN often employ entropy-based loss functions, such as cross-entropy, to minimize prediction errors during training by measuring the divergence between predicted and true node labels. Models such as CasCN [17] and AECasN [18,19] further explore spatiotemporal fusion through subgraph sequences and autoencoders [20].

Although the end-to-end models show strong performance, they still face several key challenges: First, there is a lack of deep integration between dynamic temporal sequences and local structural evolution. Second, cross-level interaction is insufficient. Third, generalization to sparse, long-tail cascades is limited. Fixed-parameter models are prone to noise and fail to adapt to high variance across samples.

To overcome these limitations, we propose the Cascading Dynamic attention-calibrated Graph Convolutional Network, named CasDacGCN. The model incorporates a bidirectional cross-attention module to align local structural cues with global temporal evolution in latent space, effectively mitigating the decoupling of spatial and temporal signals. Furthermore, we introduce a hypernetwork-based calibration mechanism that dynamically adjusts feature representations at the sample level, enhancing robustness against sparsity and long-range degradation during long-term prediction.

- We propose CasDacGCN, a dynamic attention-calibrated graph convolutional network for information popularity prediction. Our model introduces the Bidirectional Cross-Attention Fusion (BCAF) module, which dynamically integrates snapshot-level local structural features with global temporal context, mitigating the decoupling of spatial and temporal signals, and thereby better capturing the evolution of information cascades.
- We propose a Hypernetwork Calibration Module to address the challenge of sparse and long-tail cascades. It employs a lightweight feedforward network to generate sample-specific scaling coefficients, and when combined with the GCN–GRU backbone, this adaptive calibration ensures robustness across diverse cascade densities, enabling the model to generalize better than existing spatiotemporal approaches.
- Experimental results on the Weibo and DBLP datasets validate the effectiveness of our approach, demonstrating that the CasDacGCN model outperforms existing baseline methods in information propagation prediction tasks. Ablation studies further confirm the critical roles of the Bidirectional Cross-Attention Fusion (BCAF) and Hypernetwork Calibration (HFCM) modules in improving model performance.

The remainder of this paper is structured as follows. We provide a review of related works in Section 2, introduce our proposed method in Section 3, describe datasets and experimental settings in Section 4, report results in Section 5, and conclude in Section 6.

## 2. Related Work

### 2.1. Traditional Methods

Traditional approaches to popularity prediction fall into the category of feature-based methods and generative models. Feature-based methods extract signals from historical data—such as content attributes, user profiles, structural topology, and temporal dynamics—and feed them into machine learning models like regression or classification [21,22,23,24]. However, because these methods rely heavily on handcrafted feature design, they often fail to capture the complexity of large-scale and dynamic networks, which limits their generalization ability.

For content features, studies have shown that text length, hashtag patterns, and semantic relevance significantly influence repost behavior. Non-textual signals like hyperlinks, emojis, and visual features also affect content popularity. User features, such as follower count, user activeness, and social influence, have been widely utilized. Structural features describe the cascade’s network topology—depth, breadth, and early-stage shape—all of which correlate with final popularity. Temporal features capture diffusion dynamics, with many works identifying temporal patterns, burst intensity, and time-aware embeddings as key factors.

Generative models treat cascade diffusion as a temporal point process. Poisson processes assume independent events with fixed rates, while Hawkes processes incorporate self-excitation, modeling how past events influence future ones. Applications of Poisson-based models include early-stage cascade modeling and decay-aware variants. Hawkes-based models extend to time-slice prediction, brand popularity, hybrid epidemiological frameworks, and even neural implementations like Hawkesformer [25]. Despite their mathematical elegance, these models often rely on strong assumptions and struggle in complex real-world environments.

### 2.2. Deep Learning-Based Prediction Methods

The rise of deep learning has transformed popularity prediction by enabling end-to-end spatiotemporal modeling. Recurrent models such as RNNs and GRUs excel at capturing temporal dependencies, while GNNs automatically learn structural patterns from cascade graphs, overcoming the limitations of manual feature design. Entropy can quantify the uncertainty in GCN outputs, aiding in tasks like confidence estimation or structural analysis of graphs for improved decision-making. Notable models include DeepCas [26], which generates random walk sequences and encodes them via Bi-GRU with attention, and DeepHawkes [27], which integrates RNNs with Hawkes processes to jointly model event triggers.

To address incomplete topologies and long-range dependencies, hybrid architectures have been proposed. MINDS [28] optimizes multi-scale diffusion prediction through serialized hypergraphs and adversarial learning. Casformer [29] uses adaptive cascade sampling and graph transformers to predict the popularity of information propagation in social networks. MUCas [30] integrates multi-scale capsule networks and influence-aware attention, while HeDAN [31] constructs heterogeneous diffusion graphs to capture complex multi-relation influence. Other approaches incorporate dynamic routing [32] for efficient temporal encoding.

Recent advancements have also leveraged Transformer-enhanced Hawkes processes [25], autoencoder-based structural learning [22], temporal convolutional networks [33], and degree-distribution-aware deep neural networks to further enhance cascade prediction. These models demonstrate state-of-the-art performance, though challenges remain in handling sparse or incomplete network structures. We also note a broader trend across learning systems: multi-scale feature fusion and adaptive alignment between local and global signals can enhance robustness in disparate domains (e.g., AI-generated image detection) [34], echoing our use of BCAF for local–global alignment and HFCM for sample-level calibration.

In summary, existing methods are limited by handcrafted feature dependence, oversimplified generative assumptions, or insufficient structural–temporal integration. In contrast, CasDacGCN overcomes these challenges by integrating a direction-aware GCN with BiGRU for joint structural and temporal modeling, introducing the BCAF module to align local and global signals, and employing HFCM to adaptively calibrate features at the sample level, thereby achieving improved robustness and generalization.

## 3. Preliminaries

The task of modeling information cascade prediction is relatively challenging. To address this, cascade graphs are commonly adopted, as they retain adequate topological characteristics required for prediction. For clarity, this section begins with definitions and explanations of relevant concepts before introducing the proposed model. The relevant symbols are shown in Table 1.

**Definition** **1** **(Cascade Graph).**  *Let G=(V,E) represent a static social network, where V denotes the set of all nodes (users) and E denotes the set of edges. For each message i disseminated within the network, an information cascade is formed. The corresponding cascade graph is defined as the subgraph $C_{i}=\left(V_{i},E_{i}\right)$, where $V_{i}$ refers to the set of nodes that the message passes through, and $E_{i}$ denotes the edges connecting these nodes. Since message diffusion is time-dependent, we further define $C_{i}^{T}=\left(V_{i}^{T},E_{i}^{T}\right)$ as the cascade graph of message i observed within a time window T from the beginning of its propagation.*

**Definition** **2** **(Activation State).** *A node is considered activated once it retweets a message, which is equivalent to stating that the message has reached this node. Conversely, a node is inactive if it has not been activated. At time t, if a message passes through a node, its state is assigned a value of *1*; otherwise, it is* 0*. The state of a node is denoted as* $X_{i}^{T}\left(t\right)$*, representing its status within the cascade subgraph* $C_{i}^{T}$ *at time t, where T refers to the observation window.*

**Figure 1:**
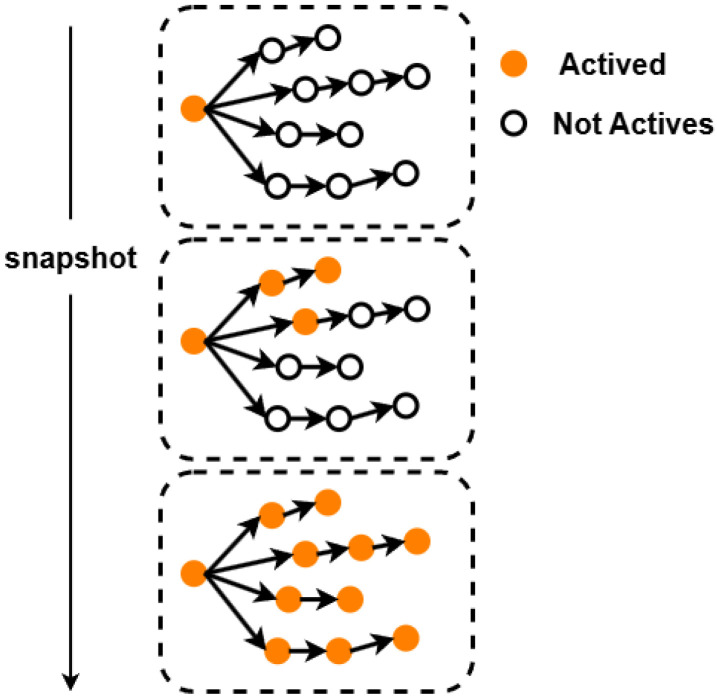
Illustration of snapshot diagrams at different time steps. Activated nodes are marked in orange, and non-activated nodes are in white.

**Definition** **3** **(Cascade Snapshot).** *As illustrated in Figure 1, only one node is marked in orange, indicating that the message has just been broadcast. All other nodes have state vectors* $X_{i}^{T}\left(t\right)$ = 0*. In the middle diagram, three additional nodes are marked in orange besides the initial sender, showing that three users have forwarded the message. The cascade subgraph*  $C_{i}^{T}$ *at this moment consists of these four nodes, each with a state of* 1*, while the remaining nodes are* 0*. In the bottom diagram, all nodes are involved in forwarding, so the cascade subgraph becomes the entire graph and the state vectors of all nodes are* 1*. By combining the cascade subgraph* $C_{i}^{T}$ *with the node states* $X_{i}^{T}$*, we obtain the snapshot* $S_{i}^{T}=\left\{V_{i}^{T},\;E_{i}^{T},\;X_{i}^{T}\left(t\right)\right\}$ *, which represents both the structure of the cascade graph and the node states at time t.*

**Figure 2:**
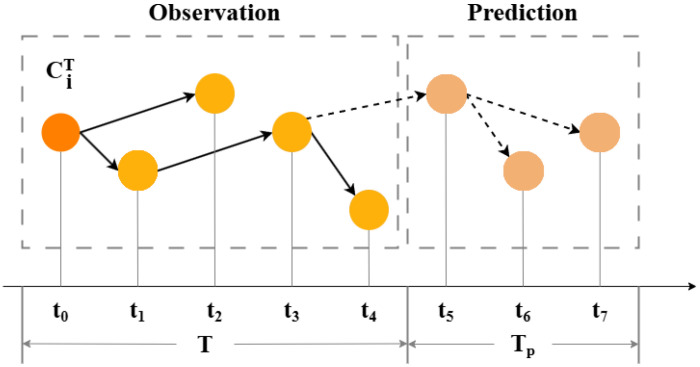
A schematic representation of the information cascade.

**Definition** **4** **(Popularity Prediction).** *Our focus lies in the cascade scale, which is characterized by the count of retweets or, equivalently, by the number of nodes (users) that a message reaches. In particular, we estimate the expansion size $\Delta R_{i}^{T_{p}}=\left|R_{i}^{T+T_{p}}\right|-\left|R_{i}^{T}\right|$ of a cascade after an observation window T, as illustrated in Figure 2.*

## 4. The Model

**Figure 3:**
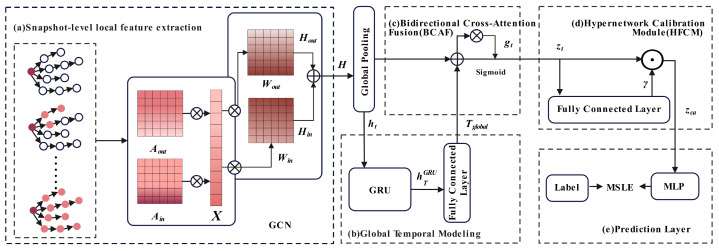
The framework of CasDacGCN.

We propose CasDacGCN, as an end-to-end framework for popularity prediction. As illustrated in Figure 3, the model consists of four interdependent modules. (a) A direction-aware GCN encoder extracts snapshot-level structural features by aggregating incoming and outgoing node information separately, capturing role asymmetry in diffusion. (b) A bidirectional GRU encodes the sequence of snapshots to model long-term temporal dependencies. (c) A cross-attention fusion module adaptively combines structural and temporal representations using a learned gating mechanism. (d) A hypernetwork-based calibrator generates sample-specific scaling factors to enhance robustness under sparse conditions. (e) Prediction layer with an MLP for log-scale popularity prediction.

### 4.1. Snapshot-Level Local Feature Extraction

The dynamic characteristics of information diffusion require the model to accurately capture the local structural evolution within each time window. To achieve this, this paper employs directed graph convolutional networks (GCNs) and global pooling operations to extract snapshot-level local features from two dimensions: directional propagation and multi-order neighborhood aggregation.

First, the snapshot partitioning and node state construction process divides the information propagation process into *T* consecutive snapshots $\left\{G_{1},G_{2},\ldots ,G_{T}\right\}$ over time, where each snapshot $G_{t}$ characterizes the propagation state within time window [t−Δt,t]. Specifically, a node state matrix $X\in R^{N\times 1}$ is first constructed, where N is a predefined fixed node count covering all potentially participating nodes. If node $v_{i}$ is activated within the window, its state is set to $X_{i,1}=1$; otherwise $X_{i,1}=0$. To unify node counts across snapshots, zero-padding is applied to newly added nodes, ensuring matrix dimensional consistency. For example, if a snapshot has M<N actually activated nodes, the remaining N−M rows are filled with zero vectors.

Next, we construct the directed adjacency matrices $A_{out}$ and $A_{in}$ to represent the outgoing and incoming edges, respectively. Both of these matrices are N×N square matrices, representing the directional information flow between nodes. Specifically, the out-edge adjacency matrix $A_{out}\left[i,j\right]=1$ if there is an edge $v_{i}\to v_{j}$, otherwise 0, and the in-edge adjacency matrix $A_{in}\left[i,j\right]=1$ if there is an edge $v_{j}\to v_{i}$, otherwise 0. Assuming snapshot $G_{t}$ contains a propagation path $v_{1}\to v_{2}\to v_{3}$, the matrices are expressed as

(1) $$A_{out}=\left[\begin{array}{ccc} 0 & 1 & 0\\ 0 & 0 & 1\\ 0 & 0 & 0 \end{array}\right],\;A_{in}=\left[\begin{array}{ccc} 0 & 0 & 0\\ 1 & 0 & 0\\ 0 & 1 & 0 \end{array}\right]$$

Traditional GCNs aggregate neighboring information symmetrically and fail to distinguish between propagation directions, which can lead to confusion regarding node roles. To address this, we employ a two-layer directed GCN that separately processes the out-edge and in-edge features. The first-layer GCN extracts low-order structural features, and its out-edge feature aggregation is given by

(2) $$H_{\mathrm{out}}=\sigma \;\left({\tilde{A}}_{\mathrm{out}}\;X\;W_{\mathrm{out}}^{\left(0\right)}\right)$$

where $W_{\mathrm{out}}^{\left(0\right)}\in R^{d\times 1}$ is the out-edge weight matrix, ${\tilde{A}}_{\mathrm{out}}$ is the normalized adjacency matrix and σ represents the ReLU activation function. The in-edge feature aggregation is expressed as

(3) $$H_{\mathrm{in}}=\sigma \;\left({\tilde{A}}_{\mathrm{in}}\;X\;W_{\mathrm{in}}^{\left(0\right)}\right).$$

Here, $W_{\mathrm{in}}^{\left(0\right)}\in R^{d\times 1}$ is the in-edge weight matrix, and ${\tilde{A}}_{\mathrm{in}}$ is the normalized in-edge adjacency matrix. The resulting feature matrices $H_{\mathrm{out}}$ and $H_{\mathrm{in}}$ both have dimensions N×d, where *d* is the feature dimension of each node.

To preserve directional information and avoid overfitting, the out-edge and in-edge features are fused by simple addition to form the fused feature representation $H_{1}$:

(4) $$H_{1}=H_{out}+H_{in}$$

where $H_{1}$ has dimensions N×d. The second-layer GCN further extracts high-order structural features by aggregating multi-hop neighbor information based on the first-layer features. Specifically, the second-layer GCN processes $H_{1}$, resulting in high-order structural features $H_{2}$:

(5) $$H_{2}=\sigma \left(W\cdot (A_{out}+A_{in},H_{1})\right)$$

where $W\in R^{d\times d}$ is the second-layer GCN weight matrix, and $A_{\mathrm{out}}+A_{\mathrm{in}}$ represents the fused out-edge and in-edge adjacency matrices. The aggregation captures the influence of a node’s multi-hop neighbors. Finally, a global mean pooling operation is applied to transform the node-level features $H_{2}\in R^{N\times d}$ into a snapshot-level representation $H_{t}$:

(6) $$H_{t}=\frac{1}{N}\sum_{i=1}^{N}H_{2}\left[i,:\right]\in R^{d}$$

At this stage, $H_{t}\in R^{d}$, which is the global feature vector for the snapshot. By compressing the spatial dimension, it preserves the statistical characteristics of node connection patterns within the snapshot while suppressing interference from noisy nodes.

### 4.2. Global Temporal Modeling

To comprehensively capture the dynamic characteristics of information propagation, we employ a global temporal modeling module over snapshot features. Specifically, we adopt a standard GRU [35] to model the hidden state sequence $\left\{s_{1},s_{2},\ldots ,s_{T}\right\}$ from inputs $\left\{h_{1},h_{2},\ldots ,h_{T}\right\}$, where $h_{t}$ is the snapshot-level representation from Section 4.1. The GRU updates are given by

(7) $$\begin{array}{cc} z_{t} & =\sigma \;\left(W_{z}h_{t}+U_{z}s_{t-1}+b_{z}\right), \end{array}$$

(8) $$\begin{array}{cc} r_{t} & =\sigma \;\left(W_{r}h_{t}+U_{r}s_{t-1}+b_{r}\right), \end{array}$$

(9) $$\begin{array}{cc} {\tilde{s}}_{t} & =\tanh\;\left(Wh_{t}+U\left(r_{t}⊙s_{t-1}\right)+b\right), \end{array}$$

(10) $$\begin{array}{cc} s_{t} & =\left(1-z_{t}\right)⊙s_{t-1}+z_{t}⊙{\tilde{s}}_{t}. \end{array}$$

where $s_{t}\in R^{d}$ is the hidden state at time step *t*, σ denotes the sigmoid function, ⊙ represents element-wise multiplication, $W_{z},U_{z},W_{r},U_{r},W,U$ are trainable weight matrices, and $b_{z},b_{r},b$ denote bias vectors. After processing the entire sequence through the GRU, the hidden state $s_{t}$ at the final time step is taken as the global temporal representation of the cascade propagation trajectory. To align with the dimensionality of local features, a linear projection layer is added:

(11) $$T_{global}=W_{proj}s_{T}+b_{proj}$$

where $W_{proj}$ and $b_{proj}$ are the learnable parameters of the projection layer. Finally, $T_{global}\in R^{d}$ represents the context vector integrating global temporal dynamics.

### 4.3. Bidirectional Cross-Attention Fusion

The snapshot-level local feature $h_{t}$ emphasizes the propagation structure within the current time window, while the global temporal feature $T_{\mathrm{global}}$ reflects the overall evolution trend of the cascade. Direct concatenation or weighted summation may lead to insufficient sensitivity to critical time points due to fixed weights. Moreover, the importance of different snapshots varies significantly across the cascade’s lifecycle, necessitating dynamic adjustment of fusion weights between local and global features. To address this, this paper proposes a gated bidirectional cross-attention fusion mechanism, which achieves adaptive calibration of local features and global temporal context through a dynamic gating strategy.

The local snapshot feature $h_{t}$ and the global temporal context $T_{\mathrm{global}}$ are concatenated on the feature axis to generate a joint representation vector $u_{t}\in R^{2d}$:

(12) $$u_{t}=[h_{t}\left|\right|T_{global}]$$

The concatenation operation preserves the independent distribution information of local and global features, preventing feature confusion, while also providing richer input for gating coefficient computation and eliminating the impact of scale differences on weight learning.

A dynamic gating coefficient $g_{t}$, quantifying the relative importance of local features in the current snapshot, is generated through a fully connected layer and a sigmoid function:

(13) $$g_{t}=\sigma \left(w^{T}u_{t}+b\right)$$

where $w\in R^{2d}$ is the learnable weight vector, *b* is the bias term, and σ is the sigmoid function.

Based on the gating coefficient $g_{t}$, a weighted fusion of the local feature $h_{t}$ and the global context $T_{\mathrm{global}}$ is performed to generate the calibrated snapshot representation $z_{t}$:

(14) $$z_{t}=g_{t}\cdot h_{t}+\left(1-g_{t}\right)\cdot T_{global}$$

Through end-to-end training, the global context $T_{\mathrm{global}}$ can be indirectly refined by the local features ${\left\{h_{t}\right\}}_{t=1}^{T}$, enabling bidirectional information interaction. Finally, the fused representations of all snapshots $\left\{z_{1},z_{2},\ldots ,z_{T}\right\}$ are aggregated along the temporal dimension to generate the cascade’s global representation:

(15) $$z=\frac{1}{T}\sum_{t=1}^{T}z_{t}$$

### 4.4. Hypernetwork Calibration Module

Different samples exhibit significant variations in propagation patterns, making fixed-parameter models difficult to generalize. Directly using uncalibrated global feature representations may render the model sensitive to sparse data or noise. Motivated by this challenge, this paper presents a Hypernetwork-based Feature Calibration Module (HFCM), which dynamically generates sample-level calibration coefficients through a lightweight network to achieve adaptive adjustment at the feature dimension. The HFCM generates sample-specific calibration coefficients via a lightweight hypernetwork and applies them to rescale the fused representation. Intuitively, in sparse cascades with very few reposts, the calibration coefficients tend to emphasize global temporal information to mitigate the lack of structural signals; in dense cascades with many reposts, they place more weight on structural cues, allowing the model to fully exploit rich local interactions. For clarity of positioning, our hypernetwork is conditioned on the post-fusion cascade representation z (from the bidirectional cross-attention module), rather than on raw inputs or a single branch, and it outputs a dimension-wise bounded vector $\gamma \in {\left(0,1\right)}^{d}$ (instead of a single scalar gate), enabling fine-grained calibration under long-tail and sparse settings. The scaling is applied at the global representation stage—after fusion and before the MLP predictor—via Equation (17), without altering layer-internal normalizations; the module is trained end-to-end under the same MSLE objective as the predictor, adding no auxiliary losses. Instead of fixed parameters, the hypernetwork generates input-dependent calibration coefficients. Taking the cascade’s global representation $z\in R^{d}$ as input, it employs a single-layer fully connected network with a sigmoid activation function to produce dimension-wise calibration coefficients $\gamma \in {\left(0,1\right)}^{d}$:

(16) $$\gamma =\sigma \left(W_{\mathrm{calib}}z+b_{\mathrm{calib}}\right)$$

where $W_{\mathrm{calib}}\in R^{d\times d}$ is the weight matrix, $b_{\mathrm{calib}}\in R^{d}$ is the bias term, and σ denotes the element-wise sigmoid function, ensuring $\gamma_{i}\in \left(0,1\right)$ for all elements in γ.

After obtaining the calibration coefficient vector, the module performs element-wise adjustment on the input holistic representation z, computing the calibrated representation $z_{\mathrm{ca}}$ as

(17) $$z_{\mathrm{cal}}=\gamma ⊙z$$

where ⊙ denotes element-wise multiplication. Through this operation, each dimensional feature value is scaled according to its corresponding calibration coefficient, enabling the model to adaptively amplify or suppress information across specific dimensions. This enhances both the robustness of overall feature representation and the predictive capability of the model.

### 4.5. Prediction Layer

After completing local snapshot feature extraction, global temporal modeling, bidirectional cross-attention fusion, and hypernetwork calibration, the model obtains the calibrated global cascade representation $z_{\mathrm{cal}}\in R^{d}$, which integrates local structural patterns, temporal dynamics, and sample-specific calibration. To map this *d*-dimensional vector to the prediction target, we employ a multilayer perceptron (MLP) head $f_{\theta }:R^{d}\;\to \;R$ that outputs a scalar:

(18) $$\hat{y}=f_{\theta }\left(z_{\mathrm{cal}}\right)\in R.$$

The cascade growth scale is defined as the increment of reposts ΔR within the prediction window $T_{p}$, with the log-transformed target

(19) $$y=\log_{2}\;\left(\Delta R+1\right),\;\Delta R=|R^{T+T_{p}}-R^{T}|,$$

where $R^{t}$ denotes the cumulative repost count within the observation interval (0,t].

During training, model parameters are optimized by minimizing the Mean Squared Logarithmic Error (MSLE) over mini-batches. For a mini-batch ℬ, with ${\hat{y}}_{i}=f_{\theta }\left(z_{\mathrm{cal},i}\right)$ and $y_{i}=\log_{2}\left(\Delta R_{i}+1\right)$, the objective is

(20) $${\text{ℒ}}_{\mathrm{MSLE}}=\frac{1}{\left|\text{ℬ}\right|}\sum_{i\in \text{ℬ}}{\left(y_{i}-{\hat{y}}_{i}\right)}^{2},$$

which is also used as the primary evaluation metric at test time.

## 5. Experiment

### 5.1. Experimental Setup and Datasets

The experimental environment of this model consists of a server running the Windows Server 2019 operating system, equipped with 128 GB RAM (manufactured by Kingston Technology, Fountain Valley, CA, USA) and an Intel Xeon Processor (Icelake) 2.59 GHz CPU (manufactured by Intel Corporation, Santa Clara, CA, USA).

In this study, we employ two datasets: the Weibo dataset for predicting repost cascades of Sina Weibo posts and the paper citation dataset for predicting academic paper citation cascades. Weibo: The dataset provided by Zhang et al. [36] was collected from the Sina Weibo platform and contains approximately 300,000 information cascade records, fully documenting the follow relationships between users and the repost chains of messages. Each repost behavior is defined as a directed edge, effectively characterizing the information propagation paths in the social network. DBLP: This dataset describes the structure of academic paper citation networks [37], constructing a directed network by parsing citation relationships between papers. Its propagation mechanism shares structural similarities with Weibo reposts.

**Figure 4:**
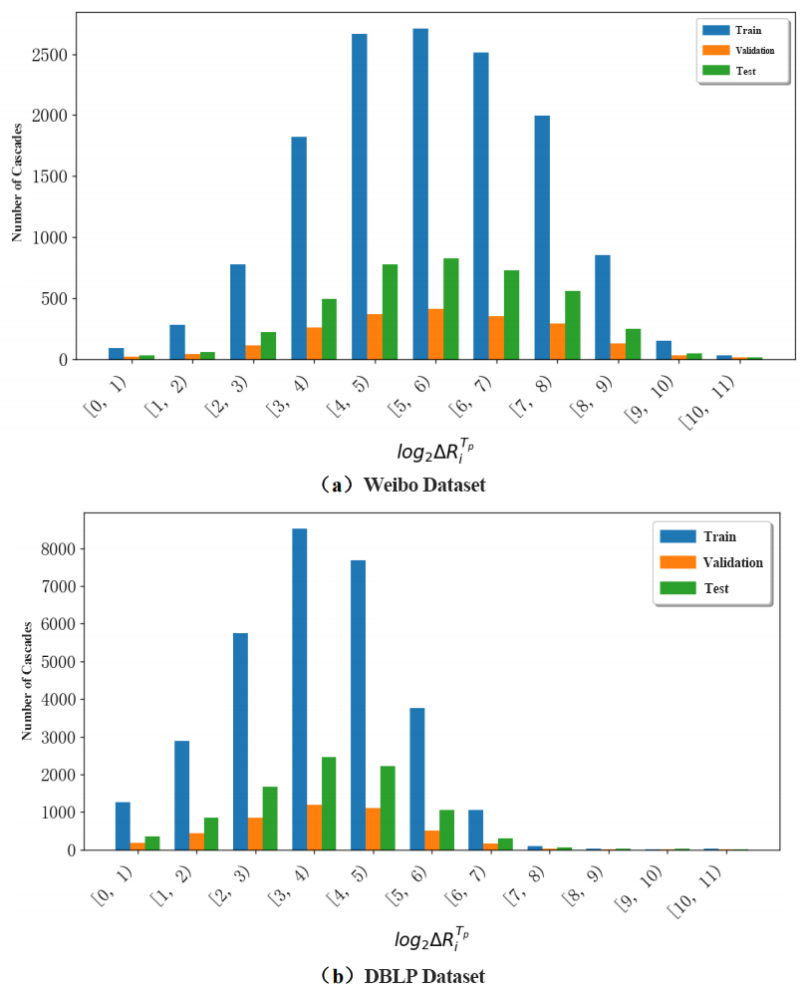
This figure shows the popularity distribution of cascades in train/validation/test splits.

For each sub-dataset, we adopt a fixed split ratio of 70% training, 10% validation, and 20% testing to ensure robust evaluation. Specifically, for the Weibo dataset (≈29,000 cascades) and the DBLP dataset (≈30,000 cascades), the corresponding splits are approximately 20,500 training, 3000 validation, and 5800 testing cascades each. As shown in Figure 4, the distributions of cascade popularity across train/validation/test splits are highly consistent, with overlapping curves and no significant distributional bias. The *x*-axis intervals [0,1),[1,2),… represent discrete bins of cascade popularity, where popularity is defined as the final number of reposts (or activated nodes). For instance, [0,1) corresponds to cascades with fewer than one repost, [1,2) to those with one repost, and so forth. This histogram-style binning provides a clear view of how cascades are distributed across different popularity levels.

Table 2 summarizes the statistical properties of the datasets. The table data statistics display the total number of nodes and edges in both the Sina Weibo dataset and the DBLP paper citation dataset. Additionally, this study provides statistics on the number of cascades, average nodes, average edges, and average popularity under different observation windows across the entire dataset. These statistical metrics visually reflect the data distribution and further provide data support for model performance evaluation. For the Weibo dataset, the observation window *T* is set to 1/2/3 h, corresponding to prediction time windows $T_{p}$ of 23, 22, and 21 h, respectively. For the DBLP dataset, three temporal subsets are constructed, with observation window *T* set to 3/5/7 years, corresponding to prediction time windows $T_{p}$ of 7/5/3 years.

### 5.2. Parameter Settings and Baselines

The model employs a two-layer GCN with hidden dimension 64, which is consistent with the output feature size of snapshots. This design balances expressive capacity and computational efficiency. In addition, preliminary experiments explored multiple learning rates {0.0001,0.0005,0.001,0.005,0.01}, and the results confirm that 0.001 yields the best convergence speed and accuracy, which aligns with prior works adopting similar settings. The GRU consists of two layers, and the hidden state dimension is configured to match the output feature dimension of the GCN. The gated fusion linear layer in the bidirectional cross-attention fusion module maintains a hidden dimension of 64, consistent with the snapshot feature dimension. The hypernetwork calibration module adopts a single-layer lightweight fully connected network to reduce computational complexity. The learning rate of the model is set to 0.001.

This paper selects several classical baseline models for comparative analysis with the proposed model. The selected baseline methods are described in detail as follows:

- Feature-linear [38]: A linear regression model is used to fit the cascade growth, with the learning rate set to 0.01.
- Feature-deep [39]: A two-layer fully connected neural network is employed to capture complex features, with the number of hidden layers set to 3 and the learning rate set to 0.001.
- DeepCas [26]: The first end-to-end deep learning model for cascade prediction.
- DeepHawkes [27]: Converts the cascade graph into forwarding paths and integrates RNN with the self-exciting mechanism of Hawkes processes for cascade prediction.
- CasCN [17]: Divides the cascade graph into subgraphs, learns subgraph representations using GCN, and models the structural evolution with LSTM.
- AECasN [18]: Employs an autoencoder to learn deep representations and outputs the predicted cascade growth.
- CasSeqGCN [32]: Generates subgraph sequences from cascade snapshots and learns structural and temporal features for prediction.
- CasDO [40] integrates probabilistic diffusion models with temporal neural ODEs to model uncertainties and irregular dynamics in cascade evolution.
- Casformer [29] introduces an adaptive cascade sampling strategy and a graph-based Transformer that jointly capture structural and temporal features of cascade graphs.

Based on existing research findings, This paper adopts the Mean Squared Logarithmic Error (MSLE) as the primary evaluation metric. This choice is motivated by the long-tail distribution and exponential growth of information cascades: while MSE and MAE are dominated by large cascades, MSLE reduces this bias through logarithmic scaling, thus enabling fairer evaluation across different cascade sizes. Moreover, this metric has been consistently adopted in prior cascade prediction studies such as DeepCas, CasFlow, CasSeqGCN, and AECasN, which ensures comparability of our results. It is formally defined as follows:

(21) $$MSLE=\frac{1}{N}\sum_{j=1}^{N}{\left(log_{2}\Delta {\tilde{R}}_{i}^{Tp}-log_{2}\Delta R_{i}^{Tp}\right)}^{2}$$

Among them, $\Delta R_{i}^{Tp}$ represents the actual repost increment of the information within the time window, $\Delta {\tilde{R}}_{i}^{Tp}$ denotes the predicted repost increment by the model, and d indicates the total number of information cascades in the dataset.

### 5.3. Performance Comparison

**Figure 5:**
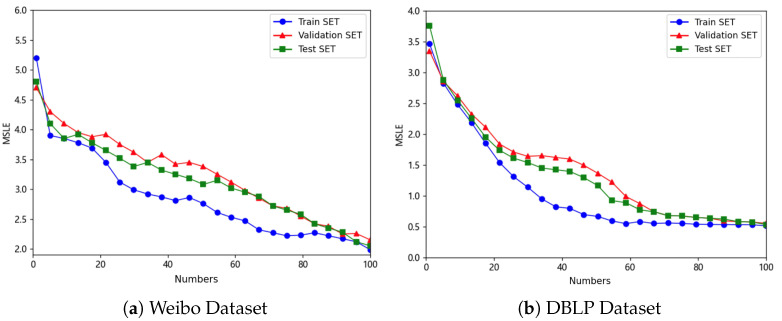
Change curve of MSLE values during the training process of the CasDacGCN model.

We first analyzed the model’s convergence speed, error variation process, and performance differences under varying observation time windows, further revealing its advantages in capturing cascading propagation characteristics. The CasDacGCN model demonstrated a robust convergence process across training, validation, and test sets. Under the conditions of a 1-h observation window for the Weibo dataset and a 7-year observation window for the DBLP dataset as shown in Figure 5, the model exhibited continuously declining error curves with significant advantages in convergence speed.

The experimental results of the CasDacGCN model and baseline methods on the Weibo and DBLP datasets are presented in Table 3 and Table 4. The experimental results demonstrate that CasDacGCN consistently outperforms all baseline methods across both datasets. On the Weibo social network, CasDacGCN achieves prediction errors of 1.985 (1 h), 1.894 (2 h), and 1.627 (3 h), yielding clear improvements over strong baselines such as CasSeqGCN (1.704 at 3 h) and Casformer (2.118 at 1 h). CasDacGCN reduces MSLE by 6.28% on Weibo-1h and 4.52% on Weibo-3h. On the DBLP academic network, CasDacGCN further demonstrates strong advantages in long-term prediction, reaching 0.517 at 7 years, which represents a 27.4% improvement over AECasN (0.712). These results verify that the bidirectional cross-attention fusion module (BCAF) effectively captures local propagation fluctuations, while the hypernetwork calibration module (HFCM) enhances the modeling of long-range sparse collaboration patterns. Moreover, when the observation time window is expanded, the predictive performance of all models improves accordingly. This improvement can be attributed to two factors: first, a larger window provides richer cascade information, enabling the directed GCN to capture second-order structural dependencies and the bidirectional GRU to extract long-range temporal patterns; second, longer propagation processes tend to stabilize, which helps establish more reliable relationships between popularity and propagation features. Through the integration of these mechanisms, CasDacGCN fully leverages additional cascade information under extended observation windows, thereby achieving more precise and robust predictions.

### 5.4. Ablation Study

To further validate the necessity and effectiveness of each module in the CasDacGCN model, comparative ablation experiments were conducted by removing different modules from CasDacGCN. Specifically, the bidirectional cross-attention fusion module (BCAF) and the hypernetwork feature calibration module (HFCM) were removed, resulting in CasDacGCN-BCAF and CasDacGCN-HFCM, respectively. The experimental setup for these comparative tests remained identical to that of the complete CasDacGCN model, using the same datasets. The experimental results are summarized in Table 5.

The experimental results demonstrate that both CasDacGCN-BCAF and CasDacGCN-HFCM models underperform the complete CasDacGCN model across both datasets, unequivocally confirming the core role of BCAF and HFCM modules. The experiments further reveal that the BCAF module is crucial for effectively capturing the interaction between local structural features and global temporal context. When removed, the model’s performance decreases significantly, particularly in long-term prediction scenarios. This highlights the importance of the dynamic alignment of local and global signals to enhance prediction accuracy. Further analysis indicates that the HFCM is designed to address the challenge of sparse and long-tail cascades by generating adaptive scaling factors for sample-specific calibration. The removal of this module leads to less stable predictions, especially when handling sparse cascades where the available information is limited. The HFCM improves model robustness and generalization by dynamically adjusting the feature representation based on the characteristics of each sample, particularly for cases with low-density cascades.

**Figure 6:**
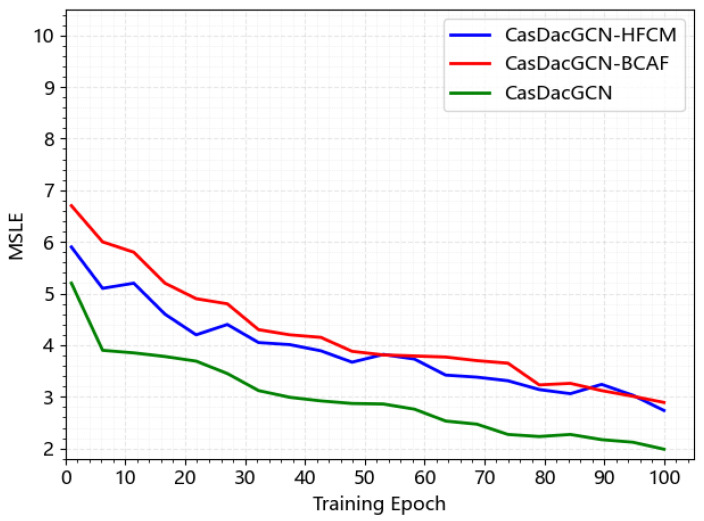
Ablation study of CasDacGCN on the 1-h Weibo dataset with different module configurations. MSLE is used as the evaluation metric.

Furthermore, to conduct an in-depth analysis of the specific performance of each comparative model in the ablation experiments, this study performed a visual analysis of the training process under a 1-h observation window on the Weibo dataset. The results are shown in Figure 6. It can be observed that the two comparative models, CasDacGCN-BCAF and CasDacGCN-HFCM, exhibit similar performance during training, with each showing relative advantages at different training epochs. The figure displays the first 100 training epochs, clearly indicating that the complete CasDacGCN model not only significantly outperforms the others in terms of error convergence speed but also achieves the optimal final error value.

### 5.5. Parameter Analysis

**Figure 7:**
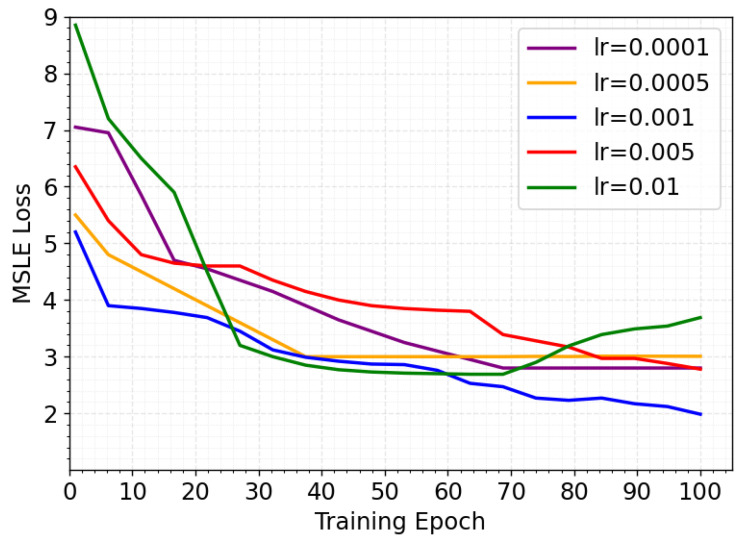
MSLE performance of CasDacGCN under different learning rates.

During the training process, this study investigated the performance of the CasDacGCN model on the Weibo dataset under different learning rates. The learning rate was selected from the set [0.0001, 0.0005, 0.001, 0.005, 0.01]. Experimental results, as illustrated in Figure 7, demonstrate that the model achieves optimal performance when the learning rate is set to 0.001, exhibiting the fastest convergence speed and the lowest error level. In contrast, lower learning rates (e.g., 0.0001 or 0.0005), while providing better convergence stability, result in slower gradient descent, requiring more iterations to reach the desired error level. Conversely, higher learning rates (e.g., 0.005 or 0.01) initially show rapid error reduction but are prone to error rebound in later stages, indicating potential overshooting of the global optimum.

## 6. Conclusions

This paper proposes a Dynamic Cascade Attention Calibration Network (CasDacGCN), which significantly improves the accuracy and robustness of information popularity prediction through direction-aware graph modeling, dynamic spatiotemporal interaction, and sparse data calibration mechanisms. Experiments validate the model’s effectiveness in social network and academic citation scenarios, demonstrating superior performance over mainstream baseline methods and providing a novel solution for dynamic network modeling. In future work, we plan to explore multimodal data sources to enrich cascade representations. These include textual content (such as post text and comments), visual features (images accompanying posts), and user profile information. We also intend to investigate causal inference approaches, including counterfactual analysis and causality-regularized learning. The goal is to better disentangle true causal effects of user interactions from spurious correlations. These directions are expected to further enhance the interpretability and robustness of popularity prediction models.

## Figures and Tables

**Table 1:** Summary of notations.

Symbol	Meaning
G=(V,E)	Static social network graph
$C_{i}=\left(V_{i},E_{i}\right)$	Cascade subgraph of message *i*
$C_{i}^{T}=\left(V_{i}^{T},E_{i}^{T}\right)$	Cascade graph observed within window *T*
$X_{i}^{T}\left(t\right)$	State of node *i* at time *t* in window *T*
$A_{out},A_{in}$	Out-edge and in-edge adjacency matrices
W,H	Weight matrix; feature matrix
$h_{t}$	Local snapshot feature at time *t*
$s_{t}$	GRU hidden state at time *t*
$T_{global}$	Global temporal representation (from GRU)
σ	Sigmoid activation function
⊙	Element-wise multiplication
z	Snapshot–temporal fused representation
γ	Calibration coefficient (from hypernetwork)
$z_{cal}$	Calibrated global representation
ΔR	Cascade growth size within prediction window
MSLE	Mean Squared Logarithmic Error

**Table 2:** Statistics of the datasets.

Dataset	Weibo			DBLP		
Obs (T)	1 h	2 h	3 h	3 y	5 y	7 y
Cascades	29.3k	30.1k	29.6k	30.1k	30.0k	30.0k
Ave Nodes	36.6	38.4	39.9	27.6	31.3	34.3
Ave Edges	30.7	32.9	33.4	33.4	44.5	50.5
Ave Popularity	184.6	128.6	101.6	30.0	16.9	8.5

**Table 3:** Experimental comparison between CasDacGCN and baseline approaches on Weibo datasets across different observation times evaluated by MSLE.

Method	Weibo		
	1 h	2 h	3 h
FeatureLinear	4.468	4.187	3.697
FeatureDeep	4.431	4.197	3.733
DeepCas	2.874	2.673	2.256
DeepHawkes	2.914	2.786	2.313
CasCN	2.722	2.574	2.203
AECasN	2.897	2.646	2.241
CasSeqGCN	2.184	2.075	1.704
CasDo	2.178	1.926	1.782
Casformer	2.118	**1.864**	1.767
**CasDacGCN (Ours)**	**1.985**	1.894	**1.627**

**Table 4:** Experimental comparison between CasDacGCN and baseline approaches on DBLP datasets across different observation times evaluated by MSLE.

Method	DBLP		
	3 Years	5 Years	7 Years
FeatureLinear	3.451	2.825	2.035
FeatureDeep	3.273	2.461	1.463
DeepCas	1.849	1.338	0.948
DeepHawkes	1.956	1.558	0.990
CasCN	1.237	1.104	0.722
AECasN	1.042	0.895	0.712
CasSeqGCN	0.884	0.735	0.527
**CasDacGCN (Ours)**	**0.806**	**0.695**	**0.517**

**Table 5:** Experimental comparison between CasDacGCN and its variants on two datasets across multiple observation times evaluated by MSLE.

Method	Weibo			DBLP		
	1 h	2 h	3 h	3 y	5 y	7 y
CasDacGCN-HFCM	2.595	2.470	2.315	1.684	1.668	1.515
CasDacGCN-BCAF	2.735	2.575	2.395	1.235	1.713	1.555
**CasDacGCN**	**1.985**	**1.894**	**1.627**	**0.806**	**0.695**	**0.517**

## Data Availability

Data are contained within the article.
